# Leveraging the developmental neurotoxicity *in vitro* battery: lessons learned interpreting assay outputs and integrating human exposure

**DOI:** 10.3389/ftox.2026.1919390

**Published:** 2026-09-24

**Authors:** Agnes L. Karmaus, Stefan Masjosthusmann, Lisa Bertomeu, Colin Ehnes, Claire Koenig, Hilary S. McCarren, Desirée Mineo, Paige Santos, Maaike E. Schutte, Bibek Sharma, Abigail C. Toltin, Kathrin Bothe

**Affiliations:** 1 Syngenta Crop Protection, LLC., Greensboro, NC, United States; 2 Bayer AG, CropScience Division, Monheim, Germany; 3 ISK Biosciences Europe N.V., Diegem, Belgium; 4 BASF SE, Agricultural Solutions, Limburgerhof, Germany; 5 Corteva Agriscience, Indianapolis, IN, United States; 6 FMC Corporation, Philadelphia, PA, United States; 7 Syngenta Ltd., Jealott’s Hill International Research Centre, Bracknell, United Kingdom; 8 ADAMA Northern Europe BV., Leusden, Netherlands

**Keywords:** developmental neurotoxicity, hazard assessment, *in vitro*, margin of exposure, NAM

## Abstract

The developing brain is highly sensitive to chemical insults due to the complexity and precise timing of neurodevelopmental processes. *In vitro* assays targeting key neurodevelopmental events offer insights into developmental neurotoxicity (DNT) pathways and represent an important advance in alternative testing strategies. The Organisation for Economic Co-operation and Development (OECD) published initial recommendations on the design and evaluation of a DNT *in vitro* battery (DNT IVB) for screening chemical effects on neurodevelopment. Thus far, there has been no characterization of the regulatory application of this new testing battery due to uncertainties in assay performance, interpretation, and contextualization of bioactivity. To gain experience with evaluating assay performance and interpretation of the DNT IVB, a subset of representative, commercially available assays were used to evaluate eight natural compounds, each with established dietary background exposure and no assumed DNT risk under “normal” dietary intake: EGCG, folic acid, vanillin, limonene, vitamin E, curcumin, genistein, and caffeine. Of the compounds tested, four were found to have “specific hits” including EGCG, genistein, caffeine, and curcumin. Concentrations at which *in vitro* specific hits occurred were compared with published human plasma levels, revealing that *in vitro* bioactive concentrations for EGCG were within relevant human exposure levels. Conversely, for curcumin, genistein, and caffeine, any identified specific hits in the evaluated DNT IVB assays occurred at concentrations higher than reported human plasma levels indicating limited relevance for human risk assessment. Moreover, our study highlights the need for well-defined performance criteria, reproducible reference controls, a standardized approach for hit calling, benchmark response determination, and interpretation across the battery to ensure reliability and reproducibility for bolstering readiness for regulatory acceptance. Overall, while the DNT IVB provides valuable insights and opportunities for screening applications to refine chemical safety evaluation, its effective use requires strengthened guidance on assay performance, data interpretation, and integration with exposure assessment to support robust application in human health risk assessment.

## Introduction

Developmental neurotoxicity (DNT) refers to adverse effects on the developing brain and nervous system resulting from exposure *in utero* through early adolescence (Henck and Morford, 2010). DNT testing is not routinely required across most global regulatory frameworks, though regional requirements differ. For example, under the European Plant Protection Products Regulation (EC) No. 1107/2009 and REACH (EC) No. 1907/2006, neurotoxicity and DNT testing are triggered when concerns are raised by chemical structure, mode of action, or existing study data. In contrast, the European Regulation for biocidal products (EC) No. 528/2012 was updated in March 2022 to introduce a standard requirement for *in vivo* DNT testing (OECD TG 443 or OECD TG 426). The US EPA requires neurotoxicity testing for all pesticides, with DNT studies mandated only on a case-by-case basis.

Exposure to neurotoxicants during critical windows of development can lead to adverse outcomes, which are currently evaluated using validated and internationally accepted *in vivo* DNT Testing Guidelines such as the OECD Test Guideline 426 (OECD, 2007), the US EPA Guideline OPPTS 870.6300 (U.S. EPA, 1998) and the OECD TG 443 (OECD, 2025a). These guidelines primarily evaluate apical outcomes, including neurohistopathology, motor and sensory function, and learning and memory. While the *in vivo* DNT assay is complex and has limitations in terms of human relevance, ethical concerns, and throughput (Juberg et al., 2023), it has served as a protective bioassay and remains the best practice and current standard.

New approach methodologies (NAMs), comprising targeted assays have advanced substantially in the area of DNT and now provide promising tools enabling higher-throughput screening of potential developmental neurotoxicants (Crofton et al., 2012; Carstens et al., 2022; Judson et al., 2010; NRC, 1984; NRC, 2007). Advances in molecular biology, cell culture techniques, and computational modeling have further paved the way for innovative assay development and analyses to refine mechanistic investigation of DNT. The OECD has published initial recommendations for the Developmental Neurotoxicity *In vitro* Battery (DNT IVB; OECD, 2023), as a novel approach for screening and prioritizing chemicals that warrant further DNT testing. Though the DNT IVB does not address all limitations of *in vivo* DNT studies, it does provide a largely human-based, targeted testing platform for single-chemical hazard assessment that can be applied within an integrated approach to testing and assessment (IATA) framework.

In its current version, the DNT IVB comprises 17 *in vitro* assays designed to screen chemical effects on the majority of known key neurodevelopmental processes (KNDPs; Bal-Price et al., 2018) utilizing an IATA framework. Specifically, the DNT IVB evaluates eight KNDPs: neural progenitor cell proliferation and apoptosis, migration of different cell types, neuronal- and oligodendrocyte differentiation, neurite outgrowth, neuronal maturation and synaptogenesis, and neural network formation. While this approach provides valuable insights and holds promise for refining chemical safety evaluations, remaining uncertainties limit its immediate application in regulatory decision making (Juberg et al., 2023). Further characterization and validation of assay performance are needed along with guidance on contextualizing results for robust interpretation. In particular, the relationship between *in vitro* changes and adverse apical neurodevelopmental outcomes *in vivo* is not yet well established, raising questions about how the DNT IVB data can be reliably integrated into regulatory frameworks.

The current study sought to contextualize DNT IVB testing outcomes by comparing *in vitro* bioactive assay potency concentrations with anticipated *in vivo* exposures levels. Eight natural compounds, each with established dietary background exposure level and no assumed DNT risk under “normal” dietary intake, were tested in a representative subset of the DNT IVB comprising the Neurosphere Assays NPC1-5 covering the KNDP cell proliferation, migration, neuronal differentiation & morphology and oligodendrocyte differentiation and the human neural network formation (hNNF) assay such that a representative assay for each KNDP (apart from apoptosis) was included. The resulting specific DNT IVB hits were then compared with human internal exposure levels to derive a margin of exposure (MOE). This MOE can be applied for a substance-specific plausibility assessment offering insight into the sensitivity of the DNT IVB and its potential utility for risk assessment.

## Materials and methods

### Test compounds

The test compounds were intentionally selected from naturally occurring substances to provide a set of chemicals with well-characterized human dietary exposures and established histories of safe consumption. This enabled contextualization of the DNT IVB results as human exposure information was available allowing interpretation of *in vitro* bioactivity relative to real-world exposure levels. The test compounds also span diverse chemical structural classes, including phenolic compounds (vanillin, curcumin), terpenes (limonene), flavanols (EGCG), alkaloids (caffeine) and compounds that infants/toddlers might be exposed to via baby formula (genistein, vitamin E, folic acid). The choice of test compounds was also driven by availability of human exposure information to put *in vitro* results in context with *in vivo* exposures. Literature searches and review of international regulatory authority assessments were conducted to derive exposure information for the individual test compounds. Information regarding the occurrence for each natural compound is summarized in Table 1.

**TABLE 1:** Natural occurrence information for the selected natural compounds^a^.

Compound	Occurrence
Caffeine	Found naturally in the seeds, fruits, and leaves of over 60 plant species, including coffee, tea, cocoa, yerba mate, and kola plants, caffeine is the most widely consumed psychoactive substance globally with an estimated 70%–80% of people consuming it daily, primarily through coffee, tea, and other caffeinated beverages (Samoggia and Rezzaghi, 2021)
Curcumin	Naturally found in the rhizome of the turmeric plant, curcumin is used in food, beverages, pharmaceuticals, and cosmetics (NCCIH, 2025)
Epigallocatechin-3-gallate (EGCG)	EGCG is the most abundant catechin found in green tea, which is a significant contributor to the global tea market, with East Asia and the Asia-Pacific regions having the highest levels of consumption (Capasso et al., 2025)
Folic Acid	Folic acid is a synthetic and more stable form of folate, which is essential for human health with a crucial role in DNA synthesis, cell division, and overall growth, especially important during fetal development. Folate is a naturally occurring B9 vitamin found in fruits, green leafy vegetables, beans, peas, eggs, and nuts. Due to its stability, it is commonly added to fortified foods like rice, pasta, bread, and some breakfast cereals (Siatka et al., 2025)
Genistein	Genistein is an isoflavone found in soy products and is commonly consumed through dietary sources like soybeans, soy milk, and tofu (Coward et al., 1993)
Limonene	A terpene found in citrus fruits like lemons, oranges, and limes; limonene is known for its distinct citrus aroma and is used as a flavor and fragrance additive in various products (Sanshita et al., 2025)
Vanillin	Vanillin (4-hydroxy-3-methoxybenzaldehyde) occurs in a wide variety of plants and is the major constituent of the natural vanilla bean. It is one of the most popular flavors worldwide, with a wide range of uses within the food, drink, cosmetic, pharmaceutical, and fragrance industries (DKFZ, 2012). It is also used by tobacco manufacturers as a flavoring material in cigarettes. As global demand far exceeds the supply that can be met by vanilla bean extraction, often alternative synthetic and biotechnological routes are used for production (Tazon et al., 2024)
Vitamin E	Vitamin E (DL-α-tocopherol acetate) is an essential vitamin naturally present in plant-derived foods such as nuts, seeds, fruits, and vegetables (NIH, 2021). It is a generic term referring to eight naturally occurring organic compounds with distinctive antioxidant activity: four tocopherols (α, β, γ, δ) and four tocotrienols (α, β, γ, δ). Among these, α-tocopherol has the highest biological activity

^a^ Next to dietary background exposure due to the consumption of plant-derived foods several of the selected compounds are also marketed as dietary supplements containing these compounds in a highly concentrated form resulting in peak exposures that may be significantly higher than the natural background exposure (see also Table 2).

All test compounds were purchased from Sigma-Aldrich; stock solutions of the test compounds were prepared in dimethyl sulfoxide (DMSO; Carl-Roth GmbH, Karlsruhe, Germany) at different stock concentrations to account for solubility limitations or cytotoxicity in range finding experiments (Table 2). A 1,000× stock solution of the test compound was prepared in DMSO. This stock was diluted 1:1000 into culture medium to obtain the highest test concentration, resulting in a final DMSO concentration of 0.1% (v/v). A series of six additional test concentrations was prepared by successive 1:3 dilutions of the highest concentration using culture medium containing 0.1% DMSO. This results in seven test concentrations with a constant DMSO concentration of 0.1% (v/v) in all treatments. Similarly, culture medium with 0.1% DMSO was used as solvent control (SC).

**TABLE 2:** Natural compounds tested in the DNT IVB and their exposure, bioavailability, and pharmacokinetic information.

Compound	Test article purity (Lot number)	Stock solution	Dietary intake information/Safe levels	Bioavailability and pharmacokinetic information
Caffeine	101% calculated on anhydrous basis (SHBN0886)	100 mM	Habitual consumption up to 400 mg/day of caffeine is considered safe for healthy adults Habitual caffeine consumption up to 200 mg per day by pregnant women does not give rise to safety concerns for the fetus or for breastfed infants exposed via milk. In pre-term neonates directly dosed with caffeine for extubation, side effects were observed at ≥ 3 mg/kg bw. Insufficient information is available to derive a safe caffeine intake for children and adolescents (Bull et al., 2015; EFSA NDA Panel, 2015a)	Clinical studies analyzing plasma concentration after single external exposure to caffeine (100–400 mg) suggest that peak plasma concentration after moderate coffee consumption can range between 7 and 80 µM (Perera et al., 2011; White et al., 2016; Willson, 2018; Cheng et al., 1990). For a 200 mg dose, a maximum peak plasma level of 44 µM was measured (Cheng et al., 1990). It can be estimated that fetal plasma levels are in a similar range, as caffeine has been shown to pass freely through the placenta (Wierzejska et al., 2014; EFSA NDA Panel, 2015a)
Curcumin	100% (97,791,015)	20 mM	The ADI for curcumin is ≤ 3 mg/kg bw/day (JECFA, 2004; EFSA ANS Panel, 2010). More specially, the EFSA panel concluded that intake from a normal European diet for both children and adults is about 0.1 mg/kg bw/day (<7% of the ADI of 3 mg/kg bw/day)	In adult humans, plasma concentrations can reach up to 1.1 ng/mL (2.9 nM) curcumin 1.5 h after ingestion (doses ranging from ∼140 to ∼2,100 mg curcumin in formulations) (Kroon et al., 2023). Adult humans administered a single oral dose of 410 mg native curcumin powder resulted in a mean Cmax of 7.1 nM (Schiborr et al., 2014)
EGCG	96% (EGCGSLCK1721)	20 mM	Each 240 mL serving of brewed green tea may provide an estimated 304 mg total catechins, with 187 mg EGCG; thus, estimated daily intake of EGCG through green tea consumption can reach approximately 560 mg/day when consuming an average of three 8 oz. Cups of green tea daily (Hu et al., 2018) In the EU, the mean daily intake of EGCG resulting from the consumption of green tea infusions ranges from 90 to 300 mg/day while exposure by high-level consumers is estimated to be up to 866 mg EGCG/day (EFSA ANS Panel, 2018) As food supplements the reported doses of tea catechins have suggested daily intakes ranging from 25 mg to 1500 mg (Hu et al., 2018; EFSA ANS Panel, 2018)	Plasma values after ingestion of daily doses of 100–1600 mg EGCG show a high variability spanning from approx. 0.04–7.4 µM (Bellmann-Strobl et al., 2021; Mukai et al., 2021; Henning et al., 2020; Scholl et al., 2018; Lorenz et al., 2017; Hu et al., 2018; Ullmann et al., 2003), with the highest bioavailability observed in humans after overnight fasting via bolus application of pure EGCG (Hu et al., 2018; EFSA ANS Panel, 2018). Rodent studies show that EGCG can reach the adult and fetal brain (Pervin et al., 2019; Chu et al., 2007; Suganuma et al., 1998). Pharmacokinetic studies in pregnant rats indicate that total EGCG is found at approximately eight times lower concentrations in the fetal brain compared to maternal plasma when administered to pregnant rats (Chu et al., 2007; Barenys et al., 2017b). Assuming similar pharmacokinetics in pregnant rats and humans, EGCG concentrations in human fetal brain of high-level green tea or EGCG supplements consuming women can be expected to reach 1 µM
Folic Acid	97% (WXBD5008 V)	20 mM	Recommended intake for people older than 19 years is 400 µg per day and if pregnant and lactating is 600 µg per day. The maximum daily dose that is unlikely to cause adverse side effects has been identified at 1000 µg/day (EFSA NDA Panel, 2023)	The reference range for internal plasma levels of folate is 2–20 ng/mL in adults (4.5–45.3 nM), 5–21 ng/mL for children (11.4–47.7 nM), and 14–51 ng/mL for infants (31.8–115.9 nM) (Fischbach and Dunning, 2015)
Genistein	99.9% (0,000,152,746)	50 mM	The background isoflavone exposure from the diet in the European population is estimated to be < 1 mg/day, however, for vegetarians or consumers of soy-based foods or supplements it could be higher (20–150 mg/day) (EFSA ANS Panel, 2015)	In adults, the mean Cmax value for genistein was 0.645 µM (435–947 nM) at 3–6 h after ingesting a soy beverage of 1.0 mg genistein (aglycone) equivalents/kg body weight (Shelnutt et al., 2002). Mean plasma concentration of genistein (including conjugates) in adult females with high protein diets containing genistein from 0.4 to 6 mg/day was 0.33–1.19 µM (van der Velpen et al., 2014). In rat studies, maternal dietary and oral gavage exposure to genistein can result in placental crossing and consequentially fetal exposure (Doerge et al., 2001; Soucy et al., 2006)
Limonene	99.4% (MKCQ8289)	100 mM	Typical background dietary intake is estimated around 660 μg/kg bw per day in Europe and 210 μg/kg bw per day in the United States of America (EFSA CEF Panel, 2011). For citrus-heavy diets estimated intakes can reach 20–40 mg/day from citrus juice and 50–90 mg/day from citrus peel (Sun, 2007)	Human limonene plasma levels at dietary background exposure were unavailable. In human cancer patients which were treated with D-limonene as potential chemotherapeutic agent, where exposure exceeds dietary levels, the Cmax was observed to be 10.8 ± 6.7 to 20.5 ± 11.2μM, after 99 courses of D-limonene 0.5–12 g/m^2^ (body surface) administered orally in 21-day cycles (Vigushin et al., 1998). In another pilot clinical study, cancer patients receiving 2 g capsules of limonene daily for 2–6 weeks were found to have serum plasma limonene concentration at an average of 0.07 ± 0.09 μg/mL in plasma (0.48 ± 0.67 μM) (Miller et al., 2013)
Vanillin	99.9% (STBK1150)	100 mM	The *per capita* intake of vanillin from its currently estimated use as a flavoring agent is approximately 0.9 mg/kg bw/day (55 mg/person/day) in Europe and 2.5 mg/kg bw/day (150 mg/person/day) in the United States of America (JECFA, 2002). Vanillin has an ADI of 10 mg/kg bw/day (JECFA, 2002) and a NOAEL of 1000 mg/kg bw/day in a 2-year study in rats (EFSA ACF Panel, 2007). Notably this NOAEL is > 100x the estimated daily intake of vanillin when used as a flavoring substance	Vanillin shows very low oral bioavailability because it is rapidly oxidized to vanillic acid, which appears in plasma within minutes while vanillin itself becomes undetectable. In humans and animals, vanillic acid is the primary circulating metabolite. It is quickly absorbed and eliminated (human T½ ≈ 1 h) and reaches a Cmax of about 2.74 μg/mL (16.3 µM) after a single vanillin ingestion of 600 mg (Singletary, 2020; Li et al., 2016)
Vitamin E	97.9% (BCCH4920)	10 mM	The EAR of vitamin E for people ages 14 and up is 12 mg/day while the RDA is 15 mg/day (Institute of Medicine DARC Institute of Medicine Panel on DARC and Vitamin, 2000), the ADI for α-tocopherol is 13 mg/day for men and 11 mg/day for women (EFSA NDA Panel, 2015b). More specifically, the tolerable upper intake level for vitamin E from all dietary sources, which were previously established by the Scientific Committee on Food, are 300 mg/day for adults, including when pregnant and lactating, 100 mg/day for children aged 1–3 years, 120 mg/day for 4–6 years, 160 mg/day for 7–10 years, 220 mg/day for 11–14 years and 260 mg/day for 15–17 years. An upper limit of 50 mg/day is established for infants aged 4–6 months and a UL of 60 mg/day for infants aged 7–11 months	In adults (including supplement users) the mean observed plasma/serum α-tocopherol concentrations are between 27 and 38 µM (EFSA NDA Panel, 2015b). In children aged 9–17 years the mean/median serum α-tocopherol concentration are between 15 and 30 µM in seven European countries. A study comparing maternal and neonatal serum α-tocopherol concentrations found that full-term neonates had approximately 5x lower mean concentrations than the birthing parent (5 µM vs. 27 μM; Wu and Chou, 2001). A human volunteer study evaluating the metabolism of vitamin E revealed baseline mean level of α-tocopherol in serum before vitamin E ingestion was 22.3 ± 7.6 μM (Radosavac et al., 2002). This concentration increased after ingestion of 306 mg of RRR-α-tocopherol and reaching a maximum at 12 h post-ingestion of 33.3 ± 11.1 μM α-tocopherol, representing an increase of about 11 μM from the baseline level. Levels returned to baseline after 72 h

Definitions: acceptable daily intake (ADI); maximal concentration (Cmax); estimated average requirements (EAR); no-observed-adverse-effect-level (NOAEL); recommended dietary allowance (RDA).

### Human neural progenitor cell (hNPC) culture

Primary human neural progenitor cells (hNPCs) from gestational week 16–19 from two donors were obtained from Lonza Verviers SPRL, Belgium. hNPCs were thawed and cultured as floating spheres in a medium consisting of Dulbecco’s Modified Eagle Medium (DMEM) and Hams F12 (both from Thermo Fisher) in a 2:1 ratio (v/v) supplemented with 2% B27 (Thermo Fisher), 20 ng/mL EGF (Thermo Fisher), 20 ng/mL FGF (R&D Systems), and 100 U/mL penicillin and 100 μg/mL streptomycin (Pan-Biotech). Neurospheres were cultured at 37 °C with 5% CO_2_, passaged mechanically with a tissue chopper (McIIwain) once a week, and half of the medium was replaced every 2–3 days. A detailed description of the test system is available in OECD initial recommendations (OECD, 2023).

### Neural network formation cell culture

SynFire glutamatergic neurons, SynFire GABAergic neurons, and SynFire astrocytes (all from NeuCyte, United States) for the neural network formation assay were thawed and cultured according to the manufacturer’s protocol and as described by Bartmann et al., 2023. Briefly, the 3 cell types were thawed and suspended in a specified proportion in seeding medium (NeuCyte). The resulting co-culture of induced neuronal (iN) and glial cells (glia) were distributed at a density of 270 x 10^3^ cells per well (140 × 10^3^ glutamatergic iN, 60 × 10^3^ GABAergic iN, 70 × 10^3^ glia) on 48 well microelectrode array (MEA) plates (Axion M768-KAP-48) or 96-well flat bottom plates (Greiner) coated with 0.1% polyethyleneimine and 20 μg/mL mouse laminin. After 24 h the cells adhered and were cultured in 250 μL short-term medium (NeuCyte). Half the medium was replaced at 3 and 5 days *in vitro* (DIV). The medium was gradually replaced to long-term medium (NeuCyte) by replacing half of the medium on DIV 7 and 10 and a complete replacement at DIV 13 (washout). Following the same plating procedure and seeding density, 20 wells of a pre-coated 96-well flat bottom plate (Greiner) were prepared for weekly cytotoxicity assessments.

### hNPC neurosphere proliferation (NPC1) assay

Spheres (Ø 0.3 mm) were plated in 96-well plates (U-bottom; 1 sphere/well) prepared with the test compound in 100 µL proliferation medium composed of DMEM and Hams F12 in a 2:1 ratio (v/v) supplemented with 2% B27, 20 ng/mL Recombinant Epidermal Growth Factor (EGF; #PHG0313; all from Thermo Fisher), 20 ng/mL Recombinant Fibroblast Growth Factor basic (FGF; #233-FB; R&D Systems), 100 U/mL penicillin and 100 μg/mL streptomycin (Pan-Biotech). Plates were incubated for up to 5 days at 37 °C with 5% CO_2_. The following controls were included on each plate: positive control for proliferation arrest (B27 medium without growth factors), BrdU background control (BG BrdU; B27 medium without addition of BrdU labeling reagent), lysis control (LC; cells lysed with Triton X100 at 0.2%, 30 min before the assay was terminated, positive control for cell viability and cytotoxicity assay) and a background control (BG; B27 medium without cells). Cell proliferation was assessed as DNA synthesis after DIV 3 using a luminescence-based bromodeoxyuridine (BrdU) ELISA (Nimtz et al., 2019). Cytotoxicity was assessed using a membrane integrity assay (CytoTox-ONE Assay, Promega) measuring the lactate dehydrogenase (LDH) release into the supernatant. Cell viability was assessed by measuring the viable cell mass using an Alamar Blue assay (Cell Titer Blue, Promega).

### hNPC neurosphere differentiation (NPC2-5) assays

Spheres (Ø 0.3 mm) were plated in poly-D-lysine (PDL/laminin)-coated 96-well plates (F-bottom; 1 sphere/well) prepared with the test compound in 100 µL differentiation medium composed of DMEM and Ham’s F12 in a 2:1 ratio (v/v) supplemented with 1% N2 (all from Thermo Fisher), 100 U/mL penicillin and 100 μg/mL streptomycin (Pan-Biotech). Plates were incubated for up to 5 days at 37 °C with 5% CO_2_. Each plate included positive controls for the respective endpoints: 0.01 mM PP2 (Src kinase inhibitor) for radial glia migration (NPC2a); 1 ng/mL EGF for neuronal differentiation (NPC3); 0.01 µM narciclasine (NCC) for neurite outgrowth (NPC4); 0.1 μg/mL Bone morphogenetic protein 7 (BMP7) for oligodendrocyte differentiation (NPC5), two lysis controls (LC; cells lysed with Triton-X100 at 0.2%, 45 min before the assay was terminated, positive controls for the cytotoxicity and viability assays); background control (BG; N2 medium without cells).

On DIV 3 (at 72 h), radial glia migration (NPC2a) was analyzed by measuring migration distance on bright field images in ImageJ. Half of the medium was replaced by newly prepared test solution in medium. The removed medium was used to assess cytotoxicity (at 72 h) based on LDH released into the culture medium (CytoTox-ONE Assay, Promega). At DIV 5 (at 120 h), cells were fixated using paraformaldehyde and stained for TUBB3 (neurons), O4 (oligodendrocytes) and Hoechst H 33258 (nuclei). The endpoint assessment was performed by high content imaging followed by automatic image analysis. Neuronal and oligodendrocyte differentiation (NPC3 and NPC5) were assessed as the percentage of all TUBB3-positive and O4-positive cells in relation to the total nuclei number within the migration area. Neurons and oligodendrocytes were automatically annotated by a machine learning algorithm (Förster et al., 2022). Subsequently, the high-content image analysis software Omnisphero was used to evaluate the migration distance of radial glia (NPC2a [120h]), neurons (NPC2b) and oligodendrocytes (NPC2c) as well as the neuronal morphology (NPC4a: mean neurite area; NPC4b: total subneurite length) (Schmuck et al., 2017; Masjosthusmann et al., 2020). Cytotoxicity was assessed as described above (CytoTox-ONE Assay, Promega). Cell viability was assessed by measuring the viable cell mass using an Alamar Blue assay before fixation (Cell Titer Blue, Promega).

Testing in the NPC1-5 assays was performed in four technical replicates for each condition and in at least three independent experiments. An independent experiment is defined as an experiment with a different cell passage or with cells from a different donor. Each compound was tested in cells from both donors.

### Human neuronal network formation (hNNF) assay

The co-culture of glutamatergic neurons, GABAergic neurons, and astrocytes in the 48-well MEA plate was exposed to the respective test compound by changing half of the medium with the respective medium containing double-concentrated compound on DIV 1, 7, 10, 14, 17, 21, 24, 28 and 31. The removed medium on DIV 10, 17, 24 and 35 was used for cytotoxicity assessment using a membrane integrity LDH assay (CytoTox-ONE Assay, Promega). 24 h before the weekly recordings at DIV 7, 14, 21, 28, and 35, a complete washout with pre-warmed PBS was performed prior to replacing the medium with chemical-free supplemented long-term medium to minimize acute substance effects during MEA recordings. The protein kinase C (PKC) inhibitor bisindolylmaleimide (Bis-I) was used as positive control (at 5 µM). Bis-I is known to decrease neurite outgrowth and firing/bursting rates of rat neural networks (Robinette et al., 2011). Bicuculline (BIC) and cyanquixaline (6-cyano-7-nitroquinoxaline-2,3-dione; CNQX) are GABAergic and glutamatergic receptor inhibitors and are used for acute pharmacological treatment of networks (at 3 and 30 μM, receptively).

Spontaneous electrical network activity was recorded with the Axion Maestro Pro system and the Axion Integrated Studio (AxIS) software version 1.5.3 or later (Axion Biosystems, Atlanta, United States). The recording procedure was as follows: 15 min of equilibration followed by two consecutive measurements of 15 min (only the last 15 min were used for analysis). For acute response measurements of positive controls, pipetting was performed directly before the measurement, with a 5 min wash-in phase and the analysis of the first 15 min of recording. All recordings were conducted at 37 °C and 5% CO_2_. The activity was measured using a gain of 1,000× and a sampling frequency of 12.5 kHz. A Butterworth band-pass filter was used (200–3,000 Hz) prior to spike detection (threshold of 6x root mean square [RMS] noise on each electrode) via the AxIS adaptive spike detector. An active electrode was defined as ≥ 5 spikes/min. The following 14 network parameters were assessed: Mean Firing Rate, Number of Active Electrodes, Number of Bursting Electrodes, Burst Duration, Number of Spikes per Burst, Mean Inter-Spike-Interval (ISI) within Burst, Inter-Burst Interval, Burst Frequency, Burst Percentage, Networkburst Frequency, Networkburst Duration, Networkburst Percentage, Number of Spikes per Networkburst and Area Under Normalized Cross-Correlation (AUNCC).

Testing in the hNNF assay was performed in three technical replicates for each condition and in at least three independent experiments. An independent experiment is thereby defined as an experiment performed with a new compound serial dilution and with cells thawed from a different vial.

### Data analysis

A detailed description of the data evaluation procedure for the NPC1-5 and the hNNF assay is available in OECD initial recommendations (OECD, 2023; Bartmann et al., 2023, respectively. Data analysis was performed with R (R Core Team, 2022)^1^ and is based on the R package CRStats a framework for the analysis of *in vitro* data that relies on the established R packages *drc* (version 3.2.0; Ritz et al., 2015) and *mvtnorm* (version 1.1–3^2^; Genz et al., 2020) paired with the step-down multiple test procedure of Dunnett and Tamhane (Dunnett and Tamhane, 1991). For both the Neurosphere Assays and the hNNF assay, the best curve-fitting model (based on AIC) for the respective data set was chosen from a large pool of 13 mathematical concentration-response functions according to robust statistical criteria. Fitted curves can follow linear, sigmoidal, monotonic, and non-monotonic trends.

All data are presented as the mean of at least 3 biological replicates ± standard error of the mean (SEM) and a p-value ≤ 0.05 was considered significant. Furthermore, the packages were adapted for concentration-response data analysis calculating the Benchmark response (BMR), lower and upper benchmark concentration (BMCL, BMCU, BMC), and the bands of the 95% confidence interval. BMR definition for each endpoint was based on the variation of the lowest compound concentration (SC-normalized) over the entire data set.

All data are provided in Supplementary Tables S1–S5 including the data of single replicates of all independent experiments from all assays (Supplementary Tables S1–S4) and summary BMC analysis results with benchmark concentration (BMC) modeling results with upper/lower confidence limits, benchmark response (BMR) values, curve trends and specificity classification (Supplementary Table S5).

### Data interpretation

For data interpretation a classification model is applied as developed by the assay developer (OECD, 2023) that classifies the result on the single assay endpoint level into one of the following categories:

Specific hit: The BMC of the DNT-specific endpoint is lower than the BMC of the unspecific (cytotoxicity/viability) endpoint and the respective BMC’s 95% confidence intervals do not overlap. In case the unspecific endpoint shows no activity, the BMCU of the specific endpoints needs to be in the tested concentration range +10% (extrapolation) or the highest concentration needs to be affected in a statistically significant (p-value <0.05) manner.
Borderline hit: (1) The BMC of the DNT-specific endpoint is lower than the BMC of the unspecific endpoint and the confidence intervals overlap by ≤ 10%. (2) There is no activity in the unspecific endpoint, the BMCU of the specific endpoint is outside the tested concentration range + 10% (extrapolation) and the highest concentration was not affected in a statistically significant (p-value <0.05) manner.
Unspecific hit: The BMC of the unspecific (cytotoxicity/viability) endpoint is lower than the DNT-specific endpoint or the confidence intervals overlap by >10%.
No hit: The compound shows no activity below the pre-defined BMR in the DNT-specific and unspecific (cytotoxicity/viability) endpoint.

The overall categorization of a compound is a hierarchical approach, based on the principle of precaution. Thereby a “specific hit” takes precedence over a “borderline hit”, which in turn supersedes an “unspecific hit”, which in turn supersedes a “no hit”. If at least one endpoint is defined as “specific hit”, the compound falls in the same category. The rationale behind this is to provide a conservative estimate of a potential hazard, under the assumption that any alteration in a key neurodevelopmental event could be a DNT concern.

## Results

### Natural compound literature review

Published scientific literature and international regulatory authority assessments were reviewed to gather information about each natural compound. Specifically, information regarding exposure levels, bioactivity, any pharmacokinetic information retrieved, and purity of samples obtained for this study are provided in Table 2. The pharmacokinetic information and exposure levels were used for helping with testing concentration range setting and later conduct of MoE calculations.

In addition to exposure and pharmacokinetic information, brief summaries of published DNT hazard/risk information were also summarized as background reference to provide further insight on these natural compounds within the context of the current testing (Supplementary File S1).

### DNT IVB assay performance

Data obtained from DNT IVB testing were first reviewed for endpoint specific positive control (ESPC) data and for replicate consistency as measures of assay performance. Reviewing control chemicals data is critical to confirm the performance of the test run and highlight the necessity of positive control chemicals for endpoint confirmation and assay responsiveness. Not all ESPCs elicited a robust response (Figure 1); in fact, some ESPC did not reproducibly affect the respective endpoint below/above the pre-defined benchmark response (30% or 50% depending on the assay endpoint). This high variability in responses was also evident in some of the test compound results. Examples are shown for caffeine in the NPC5 assay and for EGCG in the hNNF assay (Figure 2) to emphasize the challenge that variability across replicates can pose, whether in dynamic range or completely different direction of concentration-response, respectively.

**FIGURE 1:**
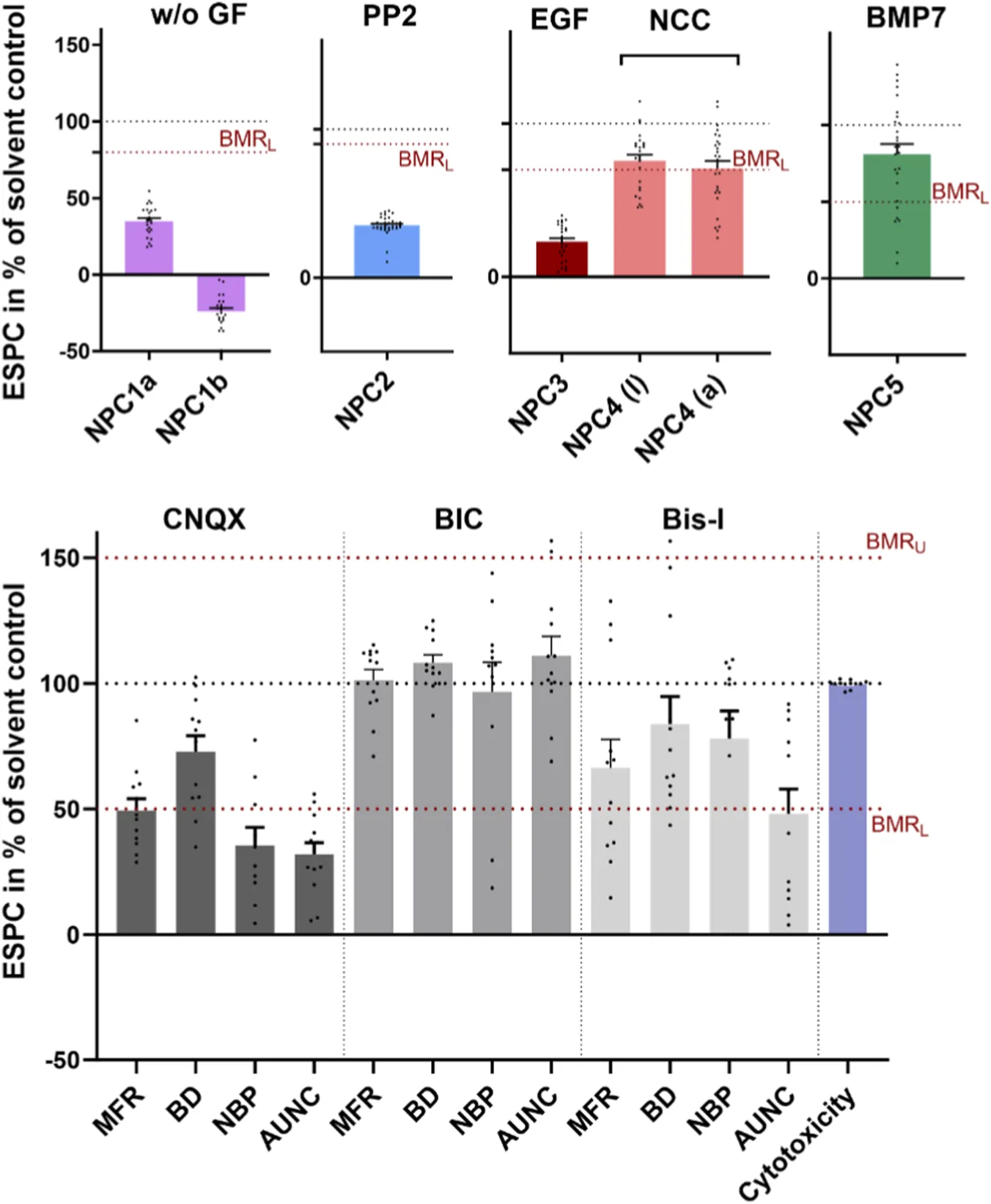
Endpoint specific control performance. Endpoint specific positive controls (ESPCs; labeled at top of figure) were evaluated relative to solvent control (0.1% DMSO) to assess dynamic range of response and confirm induction in each endpoint of the NPC1-5 assay (named above the top plot) and in four parameters in the hNNF assay (named along the X-axis in the bottom plot). Assay endpoint definitions: NPC1a/b: NPC proliferation (brdU/area; purple); NPC2: radial glia migration (cyan); NPC3: neuronal differentiation (red); NPC4 l/a: neurite length/area (pink); NPC5: oligodendrocyte differentiation (green); hNNF: human neuronal network formation (grey scale); MFR: mean firing rate; BD: burst duration; NBP: network burst percentage; AUNC: area under normalized cross-correlation. ESPC definitions: w/o GF: without growth factors; PP2: src family kinase inhibitor; EGF: epidermal growth factor; NCC: narciclasine; BMP7: bone morphogenic protein 7; CNQX: cyanquixaline; BIC: bicuculline; Bix-I: bisindolymaleimide I; BMR: benchmark response (BMR_U_ and BMR_L_ represent upper and lower bounds and were defined for each endpoint based on the variation of the lowest compound concentration (SC-normalized) over the entire data set).

**FIGURE 2:**
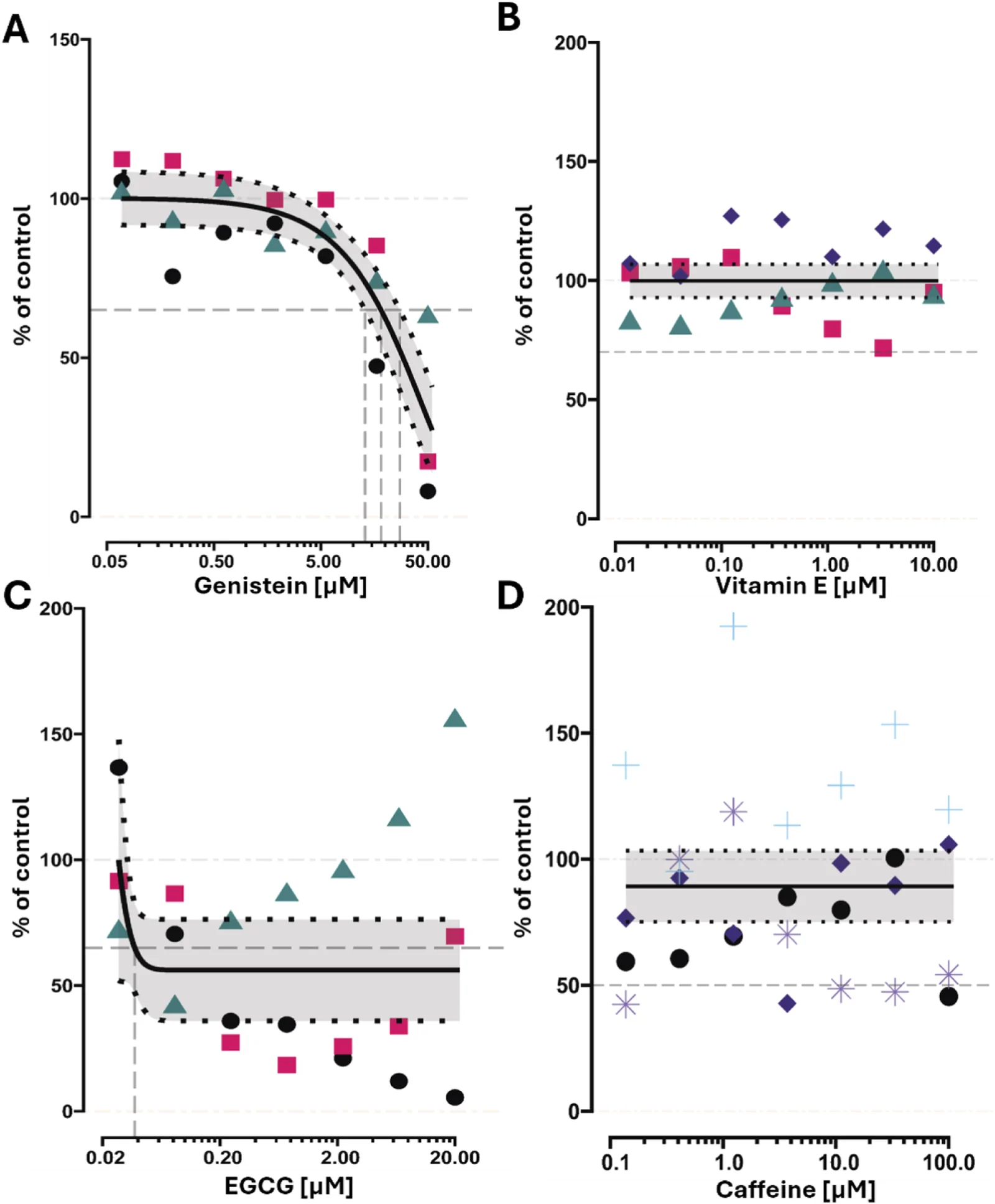
Example concentration-response data. **(A)** Genistein in the hNNF assay (AUNC: area under normalized cross-correlation) showing a concordant response across three experiments with no response in cytotoxicity (Supplementary Material 2). This response is interpreted as a specific hit call. **(B)** Vitamin E in the NPC4 assay without a response across three experiments, interpreted as a negative hit call. **(C)** EGCG in the hNNF assay (AUNC) showing discordant concentration-response trends among the 3 independent experiments, as denoted by the various shapes plotted. The winning curve model suggests an overall decreasing effect resulting in an active hit call conclusion. **(D)** Caffeine in the NPC5 assay shows inconsistent results among the 4 independent experiments. While most data points appear non-responsive, the response for one experiment is almost consistently greater than 100% lending ambiguity to whether this is a saturated response or no response. In general, the data for caffeine are considered “no hit” conclusions. Legend: different shapes and colors of the data points represent concentrations response data from independent experiments (at least three), the black line represents concentration response curve of the winning model, the dotted black lines and grey area represents the 95% confidence of the winning models curve fit, the dashed grey horizontal lines represent the BMR of the respective endpoint and the dashed grey vertical lines represent the BMC with the 95% upper and lower confidence limits.

Despite some highly variable responses, there were also very consistent and robust results as well. For example, genistein in the hNNF assay resulted in a very consistent response (despite ECGC in the same assay having highly variable results; Figure 2). This finding emphasizes the need for careful review and selection of ESCs, and that noisy data may simply indicate non-specific effects in a given assay rather than compound-specific interactions; and non-specific results for some tested compounds does not infer inability to detect robust signal overall. When compounds are clearly inactive or active, the signals are likely consistent and robust–it is these concentration-response curves that yield interpretable and reliable results. However, clear guidance is needed for individual assays regarding the interpretation of variable results.

### DNT IVB natural compound testing

Preliminary range-finding experiments conducted with testing up to 100 µM or the solubility limit (Supplementary File S2) were conducted to confirm no overt cytotoxicity was elicited by the natural compounds in the test systems. No cytotoxicity was observed for any compound in the hNNF assay nor for folic acid, vanillin, limonene, vitamin E, or curcumin in the NPC assays. Due to cytotoxicity and strongly reduced viability observed upon exposure to 100 μM EGCG and 100 μM genistein in the NPC assays range finding experiments, 20 μM EGCG and 50 μM genistein were selected as the highest tested concentrations for the definitive concentration-response testing.

Cytotoxicity and cell viability were re-evaluated in the definitive concentration-response experiments (Supplementary File S2), and the range-finding observations were confirmed. Briefly, there was no cytotoxicity observed for any compound at any testing concentration in the hNNF assay. In the definitive NPC experiments there was no impact on cell viability/cytotoxicity under proliferating conditions. However, under differentiating conditions (120 h) EGCG (at 6.7 and 20 µM) and curcumin (at 20 µM) reduced viability below the 80% cutoff and genistein (at 16.7 and 50 µM) elicited cytotoxicity and decreased viability (72 and 120 h).

After integrating cytotoxicity (e.g., identifying specific hits) and resolving replicates per assay, only four of the natural compounds had active hit call(s), considering specific hits and borderline hits, in at least one assay endpoint (Figure 3). Caffeine increased only one assay endpoint in the hNNF assay (network burst frequency) with a BMC of 86.41 µM. Genistein and curcumin had the most hits among the hNNF assay endpoints (active in 7 and 9 of 16 endpoints, respectively); however, the most sensitive effects occurred in the NPC2a assay (radial glia migration) for both. In fact, curcumin was active in the NPC2a for both timepoints (72 and 120 h) at BMCs of 8.4 and 6.1 µM. EGCG was active in the greatest diversity of assay endpoints covering several assay platforms (NPC2, NPC4, hNNF) with the lowest BMCs of all compounds tested, generally below 2 µM.

**FIGURE 3:**
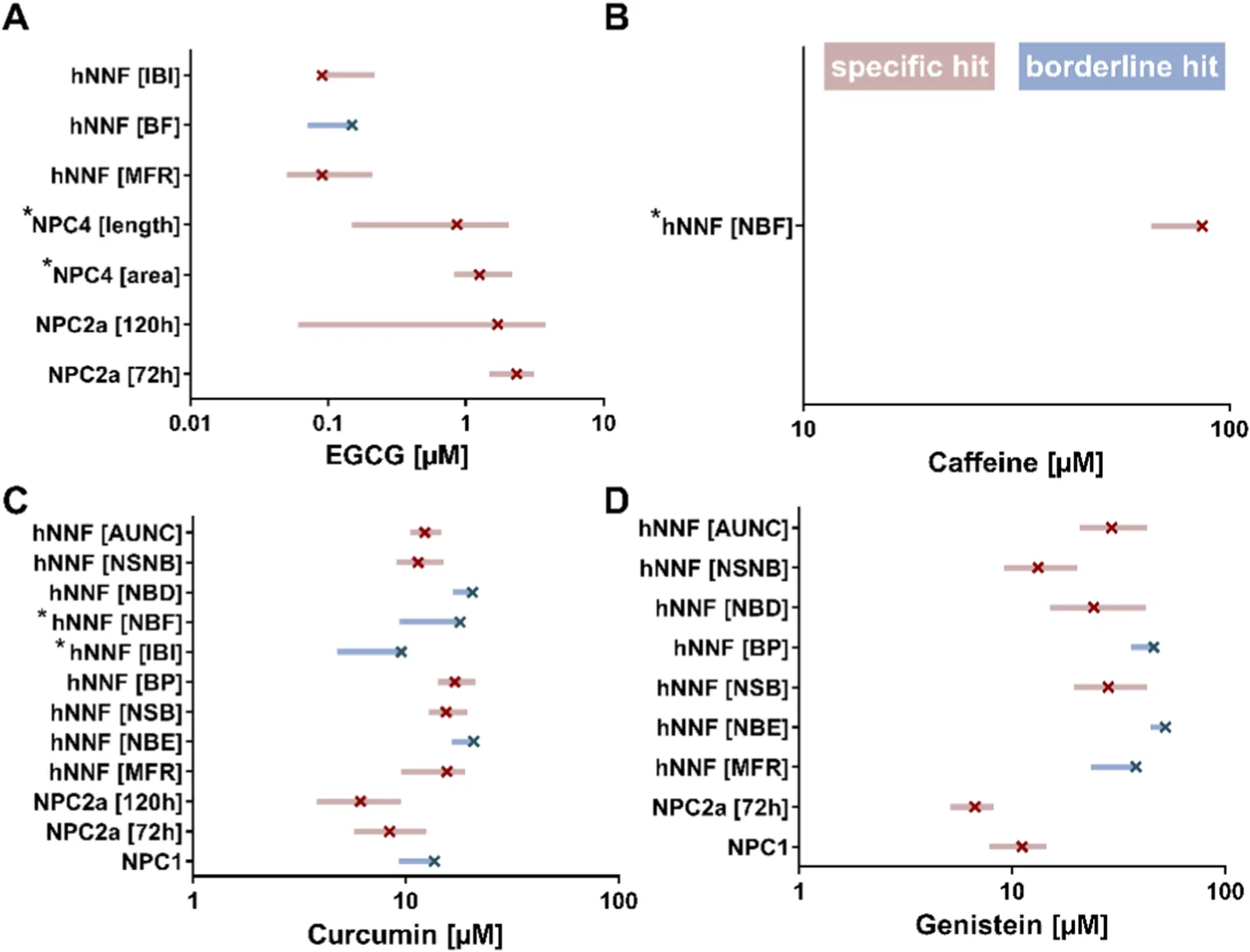
Summary of compound activity (hits) across evaluated DNT IVB endpoints. For each compound **(A)** EGCG, **(B)** Caffeine, **(C)** Curcumin, **(D)** Genistein), the BMC value per active assay endpoint is plotted with an “x”. The range (bars) represents the confidence interval for each BMC. For most endpoints a decreasing signal indicating an inhibitive effect was measured; any inductive effects are marked with an asterisk (*) next to the assay endpoint name. A borderline hit is plotted in blue; a specific hit is plotted in red. Assay endpoint definitions: NPC1: NPC proliferation; NPC2a: radial glia migration; NPC4: neurite length/area; hNNF: human neuronal network formation with parameters: IBI: inter-burst interval, BF: burst frequency, MFR: mean firing rate; AUCC BD: burst duration; NBP: network burst percentage; NBE: number of bursting electrods; NSB: number of spikes per burst; BP: burst percentage; NBF: network burst frequency; NBD: network burst duration; NSNB: number of spikes per network burst; AUNC: area under normalized cross-correlation.

The *in vitro* bioactivity BMCs were compared against human plasma levels to integrate real-world exposure context (Figure 4). The overlap shown for the tested concentration ranges and documented human plasma levels confirm that our study design encompassed *in vivo* relevant exposure ranges for each natural compound. The assay concentration range for vitamin E was limited by solubility of the test article, but with the anticipated exposure for neonates being approximately 5x lower than adults (Wu and Chou, 2001), coverage of realistic exposure levels for the most relevant human population was achieved.

**FIGURE 4:**
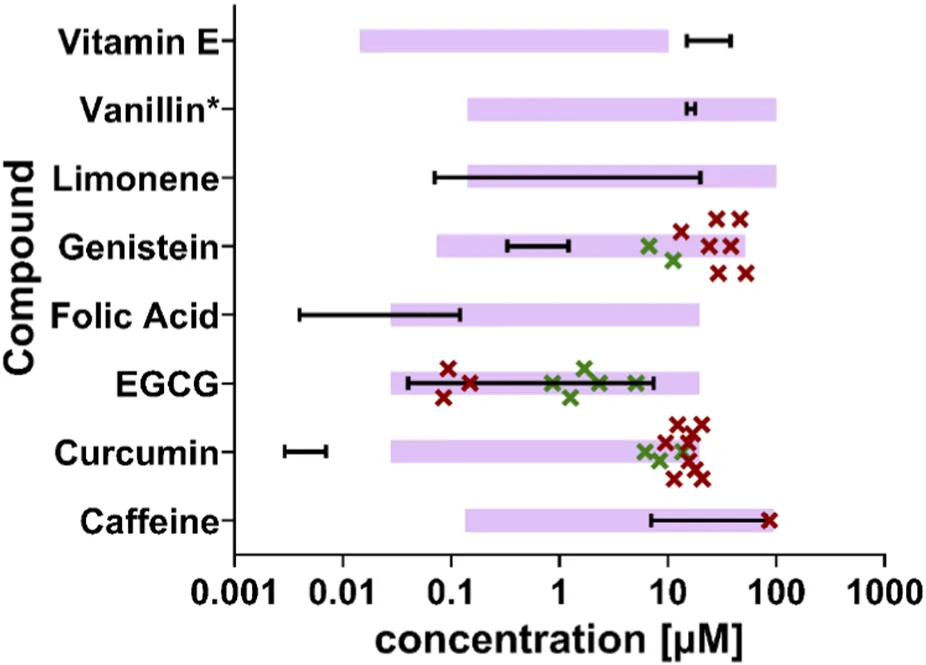
Activity of natural compounds in the DNT IVB vs. human internal exposure levels. Natural compounds BMCs including specific and borderline hits from tested DNT IVB assays (NPC1-5 endpoints in green, hNNF endpoints in red) plotted relative to the tested concentration range (purple bar) and the human exposure levels (black bars; maximum adult plasma levels from published clinical trials (see Table 2 for details on bioavailability; *plasma level of vanillin are based on vanillic acid as main metabolite).

EGCG showed *in vitro* bioactivity at the lower end of the human exposure range, suggesting bioactivity across much of the reported human *in vivo* exposure range. Caffeine showed some overlap of the reported plasma concentrations with the high end of the tested concentration range with the one bioactive endpoint landing at the very top of the distributions. Meanwhile, curcumin and genistein showed *in vitro* bioactivity only above reported human exposure levels. Overall, these findings highlight the importance of establishing appropriate concentration testing range and contextualizing *in vitro* bioactivity with human exposure information for interpreting results.

### Margin of exposure (MOE)

As a proof-of-concept to render the *in vitro* findings into a risk assessment relevant context, we computed margins of exposure (MOE) and illustrate the difference between the range of *in vitro* bioactive concentrations and human plasma exposure levels. In an effort to calculate a representative “range” of MOEs, the lower bound MOE was computed using the lowest *in vitro* DNT IVB BMC and the highest reported human adult plasma level; conversely, the upper bound MOE was computed with the highest *in vitro* DNT IVB BMC and the lowest reported human adult plasma level (Table 3).

**TABLE 3:** Calculation of margin of exposure (MOE) estimates for natural compounds showing activity in the tested DNT IVB assays.

Equation for MOE	MOE lower bound	MOE upper bound
	$\frac{lowest\;in\;vitro\;BMC}{\begin{array}{c} highest\;reported\;human\\ adult\;plasma\;level \end{array}}$	$\frac{highest\;in\;vitro\;BMC}{\begin{array}{c} lowest\;reported\;human\\ adult\;plasma\;level \end{array}}$
Caffeine	$\frac{86µM}{80µM}$ = 1.1	$\frac{86µM}{7µM}$ = 12.3
Curcumin	$\frac{6µM}{0.007µM}$ = 857	$\frac{20µM}{0.0029µM}$ = 6896
EGCG	$\frac{0.09µM}{7.4µM}$ = 0.01	$\frac{5.1µM}{0.04µM}$ = 127
Genistein	$\frac{6.7µM}{1.2µM}$ = 5.6	$\frac{52µM}{0.3µM}$ = 173

The 4 compounds that showed biological activity in the DNT IVB yielded MOEs between 0.01 and 11,000 relative to reported adult plasma levels. The wide range in MOE for some of the compounds is mainly due to the variability in human plasma levels, which of course vary greatly due to the study set-up, dietary habits, and uncertainty in free/conjugated/plasma protein bound concentrations. For caffeine (based on only one bioactive endpoint) and EGCG, the MOEs based on the lowest *in vitro* BMC and highest reported plasma concentration are close to or below one, which would require additional follow-up to ensure reference values and interpretation are appropriate for this context since the reported human adult plasma levels applied were derived from known and/or recommended dietary exposure levels. However, these findings should not be interpreted as evidence of *in vivo* hazard, nor should *in vitro* bioactivity imply *in vivo* adversity.

## Discussion

As the brain undergoes complex and precisely timed developmental processes during the prenatal and early postnatal periods, it is vulnerable to insults (Wylie and Short, 2023; Costa et al., 2004). By focusing on key events in neurodevelopment, the DNT IVB can provide insights on KNDPs that chemical may interfere with during brain development. However, due to limited commercial availability, our study included a subset of the 17 DNT IVB assays. Approaches for integrating assay outcomes remain challenging when attempting to leverage these assays in a regulatory decision-making context. Based on our experience utilizing these assays for the evaluation of natural compounds, the effective use of DNT IVB assays needs more defined approaches in which performance metrics, acceptance criteria, and interpretation guidance are provided.

Predetermined performance metrics, and confirmation of ESPCs per assay, to support assay reproducibility and to standardize interpretation of results would alleviate some of the challenges faced in interpreting the data generated herein. In the absence of defined criteria, adherence to assay protocols and scientific validation of the test methods may still be insufficient to guarantee that each run produces data within an acceptable range of variability and with the precision and accuracy required (Holzer et al., 2023). Robust, reliable, and reproducible ESPCs are critical and may warrant refinement based on the performance observed among the data collected from our study. For example, the UKN assays have a defined acceptability criterion that is based on the ESPC response and guarantees that the endpoints response is reproducible within the experimental run (criterion: “A test is discarded if the positive control did not inhibit migration, e.g., if the cell number in the migration zone is ≥ 75% of control. If the negative control decreases migration ≥ 10%, the test is discarded”; OECD, 2023). Moreover, although the OECD guidance document provides a list of potential positive and negative reference chemicals for DNT, their current level of characterization is not yet sufficient to support a robust assessment of sensitivity and specificity of not only individual assays but also the battery as a whole for DNT prediction. While such compounds would be valuable for evaluating assay and battery performance, the selected natural compounds were intended to provide a practical evaluation of DNT IVB use; having well-characterized human dietary exposure allows for the application of a workflow in which exposure contextualization can be explored.

Without a formal approach for how to calculate or report the assay variability or for setting the *in vitro* BMR, users are likely to continue developing their own interpretations. Currently, *in vitro* BMRs have been set for some assays based on both expert judgement and statistical considerations as determined by the assay developer. Whether set manually or by a standardized algorithm (e.g., ToxCast pipeline (tcpl), Filer et al., 2017), uncertainty surrounds the biological basis for selecting *in vitro* BMR levels (e.g., BMR values are defined in tcpl statistically as an effect size that is higher than the biological (control) variability of the measured endpoint). It has been demonstrated that the same raw data, processed in different ways, may result in different final outcomes that may affect the readiness status and regulatory acceptance of the NAM, as an altered output can affect robustness, performance, and relevance (Blum et al., 2025). This has also been demonstrated for the outcomes of the DNT IVB (Carstens et al., 2025; Keßel et al., 2023). Based on the findings herein, replicate variability may be a good first step to identify robust positives and negatives prior to integrating cytotoxicity, deriving points-of-departure, and ultimately hit calling per endpoint.

An additional consideration for interpretation of DNT IVB data is the robustness and reproducibility of individual assay outcomes. Review of the endpoint-specific positive controls (ESPCs) demonstrated that not all positive controls consistently produced responses exceeding the predefined benchmark response, and some exhibited substantial variability across experimental runs (Figure 1). Similar variability was observed for certain test compounds, with examples shown for caffeine in the NPC5 assay and EGCG in the hNNF assay (Figure 2). Because endpoint-specific positive controls are intended to demonstrate assay responsiveness and acceptable assay performance, inconsistent positive control responses introduce uncertainty regarding the biological significance of small or inconsistent effects observed for test compounds. These observations highlight the importance of establishing transparent assay acceptance criteria, well-characterized reference chemicals, and quantitative performance metrics to assess assay robustness, reproducibility, and dynamic range. Such considerations are particularly important when interpreting isolated assay responses, evaluating specific KDNPs, or making a determination across the DNT IVB as a whole.

Once assay performance has been established, the overall hit calling approach must be defined. The OECD guidance (OECD, 2023) acknowledges that most chemicals with DNT potential are active in more than one assay. Further, the guidance indicates that, “evaluation of results across the DNT IVB will be facilitated when a consensus approach using a common set of methods and parameters to analyze data is determined.” There has been some tendency to assign an overall positive hit call for a compound if any assay yields a “specific hit” (e.g., concentration-response effect for the assay endpoint occurring without, or more potently than, a cytotoxicity effect), despite this approach not being discretely supported by the OECD guidance. However, our findings together with the observed variability in assay responses, indicate that such an approach may be overly permissive. Furthermore, beyond assay-specific “hit calling”, the current OECD document does not advise as to how analyzed results or uncertainties should be interpreted to form a conclusion regarding what would comprise an overall “*in vitro* DNT positive” compound, what would be biologically plausible, and how a conclusion would be used in human hazard and risk assessments. Lastly, it remains unclear how to substantiate an abundance of inactive assay results as sufficient to infer inactivity for *in vitro* DNT.

Biokinetics of the test compounds should also be considered to add context for *in vitro* bioactivity. Understanding *in vitro* exposure, e.g., actual analytical measurement of media and cellular concentrations in *vitro* studies, is very rarely done when screening chemicals. Ideally, single chemical assessments should be assessed for homogeneity, concentration, and stability of the test item (e.g., per GLP or GIVIMP guidance). Such analyses were not conducted for the current study due to cost, time, and lab capability restraints Further biokinetics regarding context of human exposure are also challenging as DNT effects are dependent on compound crossing the blood-placenta-barrier (BPB), partitioning through the (maturing) blood-brain-barrier (BBB), or being secreted into breast milk and further taken up and distributed within the nursing child. Considerations of whether the target organ (e.g., brain of a fetus or young child) is even expected to be exposed are critical for interpreting any potential hazard detected *in vitro*. For the current study, a conservative exposure estimation was conducted using reported total adult plasma concentrations. This approach was selected because quantitative *in vitro* to *in vivo* extrapolation (IVIVE) for the developing brain remains challenging due to limited fetal-specific kinetic data and the lack of validated models and input parameters. In addition, European Food Safety Authority (EFSA) and OECD have noted that, as a practical and conservative surrogate, the unbound concentration in fetal brain may be approximated by the maternal plasma concentrations (EFSA PPR Panel, 2025; OECD, 2025b). Both documents also acknowledge that DNT IVB assays generally provide nominal concentrations, while the biologically effective concentration (free or intracellular concentration) may differ substantially due to binding, plastic adsorption, evaporation, metabolism, intracellular accumulation. However, as long as assay-specific distribution models are not available for the DNT IVB nominal concentrations can be used as points of departure for subsequent analysis. Although this approach carries inherent uncertainty, it provides a pragmatic first-tier framework for placing *in vitro* bioactivity into the context of human exposure. Consequently, the calculated MOEs should be regarded as screening-level estimates that provide exposure context rather than quantitative measures of human risk.

Examples of exposure contextualization for *in vitro* data are becoming more widely used but remain inconsistent and highly uncertain in model parameterization and regulatory application. For example, a recent case study for the evaluation of the agrochemical deltamethrin used PBK modeling and IVIVE to help interpret DNT IVB results into context with predicted *in vivo* exposures (Maass et al., 2023). In this data-driven approach, the MOE between the *in vitro* activity concentrations and the predicted *in vivo* concentration was >10,000. Even under the worst-case assumptions (assuming deltamethrin accumulation in the fetal plasma compared to maternal plasma and accumulation in brain compared to plasma), the MOE was still ≥80. The authors thus concluded that deltamethrin concentrations in the human fetal brain are highly unlikely to reach concentrations associated with *in vitro* findings under realistic exposure conditions (Maass et al., 2023). However, there is currently no scientific and/or regulatory consensus regarding how to integrate exposure data, what the appropriate parameterization of PBPK models for DNT should be, nor what cutoff should be applied to computed MOE to consider a compound as safe based on human *in vitro* data as points of departure.

Ultimately, assessment of the DNT potential of a compound should consider all relevant *in vitro* and *in vivo* information within an integrated approach to testing and assessment (IATA) framework. For example, a compound like EGCG that demonstrates bioactivity across multiple DNT-IVB assays at relatively low BMCs, particularly when the bioactivity concentrations substantially overlap with reported human exposure concentrations, may warrant additional investigation rather than an automatic conclusion of DNT concern. Follow-up could include review of the available toxicological and mechanistic literature, evaluation of relevant *in vivo* developmental and neurodevelopmental data, and consideration of structurally or mechanistically related compounds through read-across approaches. Quantitative IVIVE and PBPK modeling may provide additional exposure context by helping to evaluate whether concentrations associated with *in vitro* bioactivity are biologically plausible under relevant human exposure scenarios. Accordingly, for each of the natural compounds evaluated in the present study, Supplementary File S1 provides a brief summary of literature review findings pertaining to published DNT hazard or risk assessments, as well as relevant developmental and neurodevelopmental toxicity information, to place the DNT-IVB findings into the context of existing evidence. This type of integrated assessment can help determine whether *in vitro* findings are consistent with existing evidence, warrant further investigation, or are insufficient to support conclusions regarding *in vivo* DNT hazard or risk.

The results presented herein demonstrate both the opportunities and the challenges of translating *in vitro* DNT IVB results into regulatory decision-making. Because the testing conducted did not include all DNT IVB assays, it does not provide the full biological coverage of the current DNT IVB as endorsed by OECD. The untested assays and associated KNDP should therefore be considered as residual uncertainty, particularly if complete DNT IVB coverage should be required in a regulatory context. Moreover, without a defined approach the interpretation of the biological significance for these *in vitro* findings to integrate the KNDP-based insights into the context of *in vivo* likelihood for adversity remains unclear.

## Data Availability

The raw data supporting the conclusions of this article will be made available by the authors, without undue reservation.

## Footnotes

1
https://www.r-project.org/

2
https://cran.r-project.org/package=mvtnorm
