# Supplementary material for: Leveraging the developmental neurotoxicity in vitro battery: lessons learned interpreting assay outputs and integrating human exposure
Source: Front Toxicol. 2026 Sep 24;8:1919390. doi: 10.3389/ftox.2026.1919390 (PMC13645332; doi:10.3389/ftox.2026.1919390)
Supplement: Supplementary file 1 [file Supplementaryfile1.docx]

**Supplementary File 1: DNT hazard and risk summaries for natural compounds**

This supplementary file provides brief summaries of literature review findings pertaining to any reported DNT hazard or relevant risk assessments for each of the natural compounds evaluated in the presented study to serve as background information.

## EGCG

Green tea preparations have been assessed by various international bodies regarding their potential benefits and risks, particularly concerning hepatotoxicity linked to high doses of green tea extracts. Uncertainties exist due to inadequate pharmacokinetic and toxicological data, especially regarding EGCG's safety during prenatal development (EFSA ANS Panel 2018). *In vitro* studies indicate neurodevelopmental toxicity potential through impaired cell migration, attributed to its binding to laminin and interaction with integrin-β1 (Barenys et al., 2017a, 2017b; Kühne et al., 2019, 2022; Peter et al., 2017; Suzuki and Isemura, 2001). Reduced number of neurons in the migration area and reduced neurite and dendritic length after EGCG developmental exposure were identified *in vitro* and *in vivo* in rodents as additionally affected endpoints (Barenys et al. 2017, Kühne et al. 2019, Kühne et al. 2022). This study confirms EGCGs effects on cell migration and neurite morphology; however, in the current set-up increased neurite length in human neurons were observed. Additionally, changes in neuronal activity at lower concentrations compared to effects reported in other *in vitro* DNT assays were shown. The fact that the derived *in vitro* BMCs overlap with published human plasma levels may suggest that further DNT assessment of EGCG and similar supplements during pregnancy may be warranted.

## Vitamin E

Alpha-tocopherol, the predominant vitamin E form actively taken up by the brain, offers neuroprotective benefits against oxidative stress, aiding in neurodegenerative disease prevention (Clement et al., 1995; da Cunha Germano et al., 2023). It also plays a crucial protective role in brain development. High maternal vitamin E intake protects offspring in rodent studies by reducing brain atrophy and DNA damage from ethanol exposure (Shirpoor et al., 2009) and mitigating fetal brain dysfunction caused by neurotoxicants like acrylamide, lead, and tobacco smoke (Erdemli et al., 2017; Antonio-Garcia and Masso-Gonzalez, 2008; Yang et al., 2008). Conversely, moderate maternal deficiency is linked to lower brain vitamin E levels at birth and permanent changes in brain chemistry and anxiety in adult mice (Desrumaux et al., 2018). Vitamin E is also required for typical neurodevelopment in zebrafish (Head and Traber, 2021). However, excessive maternal intake (1000 mg α-tocopherol/kg bw/day in rats) may cause lasting changes in synapse density and neuron-glia relationships in the hippocampus, potentially affecting cognitive behavior (Ambrogini et al., 2016). The mechanisms of these lasting effects remain unclear, with suggestions that α-tocopherol could impact PKC phosphorylation and increase glutamate uptake, leading to altered axo-spinous synapse density and neuron-glia morphological organization at synapses (Salucci, et al., 2014). EFSA concluded in 2015 that there is a lack of studies on reproductive and developmental endpoints following α-tocopherol exposure, and no multi-generational studies were conducted according to proper guidelines. Thus, it deemed the existing toxicity database insufficient to establish an acceptable daily intake for tocopherol-rich extracts and vitamin E variants (EFSA NDA Panel, 2015b). Our testing in the DNT IVB data revealed no hits, but it remains uncertain whether relevant modes of action were adequately assessed.

## Genistein

Genistein, an isoflavone from soy, may affect brain development through estrogen signaling, impacting neuronal and glial cell processes. It has been shown that contrary to estrogen, isoflavones, specifically genistein and daidzein, are toxic to primary neuronal culture at high concentrations (Jin et al., 2007). Moreover, isoflavones can induce and modulate not only various signaling pathways through estrogen signaling, but also other receptors like TR (thyroid hormone receptor) and PPAR (Ariyani et al., 2018; Patel and Barnes 2010). A recent review concluded that isoflavones’ neurodevelopmental effects depend on the timing, dose, and duration of isoflavones exposure/treatment and that adverse effects at higher doses cannot be excluded (Ariyani and Koibuchi, 2024). The EFSA Panel on Dietetic Products, Nutrition and Allergies recommended in 2014 that concentrations of isoflavones in infant formula and follow-on formulae should be kept as low as is feasible as a precautionary principle because of the inconclusive evidence for adverse effects of dietary soy isoflavones on human sexual, reproductive and neurobehavioral development, immune function and thyroid function (EFSA NDA Panel, 2014).

Genistein was classified as a specific hit compound for hNPC proliferation and radial glia migration and specifically affected several parameters in the hNNF assay. This broad biological activity is presumably a reflection of its ability to interact with numerous signaling pathways. However, the *in vitro* hit concentrations were above reported human *in vivo* plasma levels indicating no critical risk.

## Vanillin

Vanillin presents various health benefits, including antioxidant and neuroprotective effects (Iannuzzi et al., 2023; Anand et al., 2023). A NOEL of 500 mg/kg of body weight per day for ethyl vanillin was reported in a 14-week feeding study in rats and a NOEL of 1000 mg/kg of body weight per day was reported in a 2-year feed study in rats (Joint FAO/WHO Expert Committee on Food Additives report 2002). Ethyl vanillin did not have adverse effects on reproduction and developmental toxicity up to 1000 mg/kg bw in the rat in a reproductive and toxicity screening study (OECD 421). A maternal and developmental NOAEL of 500 mg/kg bw was reported for ethyl vanillin in a developmental toxicity study in the rat (OECD 414; ECHACHEM [ECHA Chemicals Database; chem.echa.europa.eu; accessed 29 Jan 2026]). However, no specific DNT endpoints were included in these studies. Vanillin was classified as a 'no hit' compound in DNT IVB testing conducted herein.

## Caffeine

Caffeine acts as a CNS stimulant, impacting neurotransmitter release and calcium uptake and release (Fiani et al., 2021). However, with regard to effects during pregnancy and brain development, knowledge gaps remain (Porciuncula et al. 2013). A systematic literature search by EFSA in 2015 (Bull et al., 2015) concluded that caffeine intake from all sources up to 200 mg per day consumed throughout the day by pregnant women does not give rise to safety concerns for the fetus. This conclusion is based on prospective cohort studies showing a dose-dependent positive association between caffeine intake during pregnancy and the risk of adverse birth weight-related outcomes in the offspring. Possible neurodevelopment adverse outcomes were not investigated in these studies and thus not considered for the assessment. A recent systematic review of 14 observational studies found mixed evidence on the link between prenatal caffeine exposure and neurobehavioral disorders in children. With seven studies suggesting potential associations and six showing no significant effects, the authors conclude that the evidence is insufficient to confirm with certainty that the prenatal caffeine exposure leads to neurobehavioral disorders (Santana et al., 2024). Caffeine also currently enjoys the reputation of being beneficial and safe for use in neonatal medicine to stimulate respiration. However, data generated in infant mice and rats suggest the need to consider whether caffeine may have harmful as well as beneficial effects on the developing brain (Yuede et al. 2013, Heise et al. 2023). There are several studies in rodents that show behavioral and neurocognitive effects after maternal caffeine intake, even at concentrations considered safe for humans (James, 2026). In summary, while certain evidence suggests that maternal caffeine consumption at levels deemed safe may negatively affect brain development, further research is required to draw definitive conclusions.

With regard to the effects of caffeine in the DNT IVB it can be concluded that caffeine has low biological activity. In this study, only one parameter of the neural network formation assay (network burst frequency) is affected at the upper end of the tested concentration range. Considering that parameters in this assay are highly correlated, a hit call in only one parameter should be interpreted with care. In addition, caffeine has already been evaluated in several other assays included in the DNT IVB, and it did not show any biological activity. These assays examine the processes neurite outgrowth, neuronal maturation, synaptogenesis, apoptosis, neural progenitor cell proliferation, and neural network formation using different test systems as compared to this study (CompTox Chemical Dashboard v2.7.0; access data: 1/29/2026; [Caffeine - Toxcast Summary](https://comptox.epa.gov/dashboard/chemical/invitrodb/DTXSID0020232)). Nevertheless, for caffeine the MOE based on the NNF assay BMC was ≈ 1, which would require additional follow-up activities to ensure appropriate interpretation.

## Curcumin

Curcumin is a “generally recognized as safe” (GRAS) compound by different regulatory bodies, displaying protective neurological effects in literature (Fadaei et al., 2025). A re-evaluation by EFSA in 2010 concluded that intake of curcumin from the normal diet amounts to less than 7% of the ADI of 3 mg/kg bw/d, and reproductive studies were reviewed (EFSA ANS Panel, 2010). Specifically, a two-generation OECD 416 study in rats (Ganiger et al., 2007) was reviewed by EFSA and a NOAEL for reproductive toxicity was determined to be 250-230 mg/kg bw/d based on decreased body weight gain the F2 generation at the high dose level No specific DNT parameters were assessed in this study; however, no clinical observations related to acute neurotoxicity were noted in any generation. In a mouse study also reviewed by EFSA, no effects on pregnancy rate, number of dead embryos, or total implants were observed in mice fed with turmeric (0.5%) or curcumin (0.015%) (Vijayalaxmi 1980). Curcumin had several specific hits for a diverse range of endpoints. However, due to its low bioavailability (MoE: 857-6896), there is a limited risk for *in vivo* adverse outcomes.

## Limonene

Limonene has no specified ADI due to its considered safety by JECFA (2005) and EFSA (EFSA FEEDAP Panel, 2015) in its current uses. It has been tested in 4-week, 13-week and 2-year oral toxicity studies in mice and rats by the NTP, but reproductive toxicity or neurotoxicity studies were not part of the NTP data package. No evidence on teratogenic and embryotoxic effects of limonene has been reported in the literature, yet some studies indicated potential adverse outcomes in offspring (Tsuji et al., 1975; Kodama et al., 1977a, b). Toxic effects reported in adult rodents include nephrotoxicity, hepatotoxicity and neurotoxicity (Ravichandran et al., 2018). Subacute oral exposure of mice to 25, 50, and 75 mg/kg of R-(+)-limonene epoxide for 14 days did not impact behavior, food, or water consumption, and did not cause death. However, when the same doses were administered via intraperitoneal injection, mice showed reduced rearing, grooming, and movement, as well as motor coordination inhibition, possibly mediated through activity at the benzodiazepine binding site of the GABA_A_ receptor (de Almeida et al., 2012). Additional studies in rodents indicated that limonene has sedative effects by influencing dopamine and serotonin activity, and it reduced increased activity from methamphetamine (Yun, 2014). Human studies showed that inhaling limonene affects brain activity, particularly in stress and anxiety responses (Sowndharajan et al., 2015; Heuberger et al., 2001). It is therefore important to further investigate the effect of limonene on the nervous system, although it is classified as a ‘no hit’ compound in this study.

## Folic Acid

Folic acid plays a significant role in neurodevelopment, particularly during pregnancy. However, the relationship between folic acid and developmental neurotoxicity is complex and has been the subject of various studies. Folic acid supplementation before and during early pregnancy is well-established in preventing neural tube defects such as spina bifida and anencephaly. This effect is attributed to its role in DNA synthesis and methylation processes critical for fetal neural development (Czeizel and Dudáset al, 1992; Seyoum Tola 2024). Generally, adequate folate levels are associated with better neurodevelopmental outcomes in children. Studies suggest that maternal folate status during pregnancy can influence cognitive function and reduce the risk of developmental disorders (Cortes-Albornoz et al., 2021, Caffrey et al. 2019). However, high levels of unmetabolized folic acid in the bloodstream could potentially interfere with the normal metabolism of other B vitamins, such as B12, which is crucial for neurological health, or result in DNA hypomethylation, epigenetic instability and abnormal gene expression (Sun et al. 2025 ). Some research indicates that excessive folate intake may have neurodevelopmental effects in animal offspring (Xu et al. 2024) and has been associated with an increased risk of autism spectrum disorders (ASDs) in humans (Gusso et al. 2023). Folic acid showed no specific or borderline hits in the selected DNT IVB assays.

# References

Ambrogini P., Betti M., Galati C., Di Palma M., Lattanzi D., Savelli D., et al. (2016). α-Tocopherol and Hippocampal Neural Plasticity in Physiological and Pathological Conditions. Int J Mol Sci. 17(12):2107. doi: 10.3390/ijms17122107.

Anand A., Khurana N., Kaur S., Ali N., AlAsmari A.F., Waseem M., et al. (2023). The multifactorial role of vanillin in amelioration of aluminium chloride and D-galactose induced Alzheimer's disease in mice. Eur J Pharmacol. 954:175832. doi: 10.1016/j.ejphar.2023.175832.

Antonio-García M.T., Massó-Gonzalez E.L. (2008). Toxic effects of perinatal lead exposure on the brain of rats: involvement of oxidative stress and the beneficial role of antioxidants. Food Chem Toxicol. 46(6):2089-95. doi: 10.1016/j.fct.2008.01.053.

Ariyani W., Iwasaki T., Miyazaki W., Yu L., Takeda S., Koibuchi N. (2018). A Possible Novel Mechanism of Action of Genistein and Daidzein for Activating Thyroid Hormone Receptor-Mediated Transcription. Toxicol Sci. 164(2):417-427. doi: 10.1093/toxsci/kfy097.

Ariyani W., Koibuchi N. (2024). The effect of soy isoflavones in brain development: the emerging role of multiple signaling pathways and future perspectives. Endocr J. 71(4):317-333. doi: 10.1507/endocrj.EJ23-0314.

Barenys M., Gassmann K., Baksmeier C., Heinz S., Reverte I., Schmuck M., et al. (2017a). Epigallocatechin gallate (EGCG) inhibits adhesion and migration of neural progenitor cells in vitro. Arch Toxicol. 91(2):827-837. doi: 10.1007/s00204-016-1709-8.

Barenys M., Masjosthusmann S., Fritsche E. (2017b). Is Intake of Flavonoid-Based Food Supplements During Pregnancy Safe for the Developing Child? A Literature Review. Curr Drug Targets. 18(2):196-231. doi: 10.2174/1389450116666150804110049.

Bull S., Brown T., Burnett K., Ashdown L., Rushton L. (2015). Extensive literature search as preparatory work for the safety assessment for caffeine. EFSA supporting publication 2015:EN-561, 98 pp. Question No EFSA-Q-2013-00419

Caffrey A., McNulty H., Irwin R.E., Walsh C.P., Pentieva K. (2019). Maternal folate nutrition and offspring health: evidence and current controversies. Proc Nutr Soc. 78(2):208-220. doi: 10.1017/S0029665118002689.

Clément M., Dinh L., Bourre J.M. (1995). Uptake of dietary RRR-alpha- and RRR-gamma-tocopherol by nervous tissues, liver and muscle in vitamin-E-deficient rats. Biochim Biophys Acta. 1256(2):175-80. doi: 10.1016/0005-2760(95)00019-9.

Cortés-Albornoz M.C., García-Guáqueta D.P., Velez-van-Meerbeke A., Talero-Gutiérrez C. (2021). Maternal Nutrition and Neurodevelopment: A Scoping Review. Nutrients. 13(10):3530. doi: 10.3390/nu13103530.

Czeizel A.E., Dudás I. (1992). Prevention of the first occurrence of neural-tube defects by periconceptional vitamin supplementation. N Engl J Med. 327(26):1832-5. doi: 10.1056/NEJM199212243272602.

da Cunha Germano B.C., de Morais L.C.C., Idalina Neta F., Fernandes A.C.L., Pinheiro F.I., do Rego A.C.M., et al. (2023). Vitamin E and Its Molecular Effects in Experimental Models of Neurodegenerative Diseases. Int J Mol Sci. 24(13):11191. doi: 10.3390/ijms241311191.

de Almeida A.A., Costa J.P., de Carvalho R.B., de Sousa D.P., de Freitas R.M. (2012). Evaluation of acute toxicity of a natural compound (+)-limonene epoxide and its anxiolytic-like action. Brain Res. 1448:56-62. doi: 10.1016/j.brainres.2012.01.070.

Desrumaux C.M., Mansuy M., Lemaire S., Przybilski J., Le Guern N., Givalois L., et al. (2018). Brain Vitamin E Deficiency During Development Is Associated With Increased Glutamate Levels and Anxiety in Adult Mice. Front Behav Neurosci. 12:310. doi: 10.3389/fnbeh.2018.00310.

EFSA ANS Panel (European Food Safety Authority Panel on Food Additives and Nutrient Sources added to Food). (2010). Scientific Opinion on the re-evaluation of curcumin (E 100) as a food additive. EFSA Journal. 8(9):1679. [46 pp.]. doi: 10.2903/j.efsa.2010.1679

EFSA ANS Panel (European Food Safety Authority Panel on Food Additives and Nutrient Sources added to Food). (2018). Scientiﬁc Opinion on the safety of green tea catechins. EFSA Journal;16(4):5239, 89 pp. doi.org/10.2903/j.efsa.2018.5239

EFSA FEEDAP Panel (European Food Safety Authority Panel on Additives and Products or Substances used in Animal Feed). (2015). Scientific Opinion on the safety and efficacy of aliphatic and aromatic hydrocarbons (chemical group 31) when used as flavourings for all animal species. EFSA J. 13(3):4053, 22 pp. doi: 10.2903/j.efsa.2015.4053

EFSA NDA Panel (European Food Safety Authority Panel on Dietetic Products, Nutrition and Allergies). (2014). Scientific Opinion on the essential composition of infant and follow-on formulae. EFSA J.12(7):3760, 106 pp. doi: 10.2903/j.efsa.2014.3760

EFSA NDA Panel (European Food Safety Authority Panel on Dietetic Products, Nutrition and Allergies). (2015b). Scientific Opinion on Dietary Reference Values for vitamin E as α-tocopherol. EFSA Journal 2015;13(7):4149, 72 pp. doi: 10.2903/j.efsa.2015.4149

Erdemli M.E., Altinoz E., Aksungur Z., Turkoz Y., Dogan Z., Gozukara Bag H. (2017). Biochemical investigation of the toxic effects of acrylamide administration during pregnancy on the liver of mother and fetus and the protective role of vitamin E. J Matern Fetal Neonatal Med. 30(7):844-848. doi: 10.1080/14767058.2016.1188381.

Fadaei M., Lahijan A.S.N., Jahanmehr D., Ahmadi A., Asadi-Golshan R. (2025). Food additives for the central nervous system, useful or harmful? An evidence-based review. Nutr Neurosci. 28(8):910-927. doi: 10.1080/1028415X.2024.2433257.

Fiani B., Zhu L., Musch B.L., Briceno S., Andel R., Sadeq N., Ansari A.Z. (2021). The Neurophysiology of Caffeine as a Central Nervous System Stimulant and the Resultant Effects on Cognitive Function. Cureus. 13(5):e15032. doi: 10.7759/cureus.15032.

Ganiger S., Malleshappa H.N., Krishnappa H., Rajashekhar G., Ramakrishna Rao V., Sullivan F. (2007). A two generation reproductive toxicity study with curcumin, turmeric yellow, in Wistar rats. Food Chem Toxicol. 45(1):64-9. doi: 10.1016/j.fct.2006.07.016.

Gusso D., Prauchner G.R.K, Rieder A.S., Wyse A.T.S. (2023). Biological Pathways Associated with Vitamins in Autism Spectrum Disorder. Neurotox Res. 41(6):730-740. doi: 10.1007/s12640-023-00674-z.

Head B., Traber M.G. (2021). Expanding role of vitamin E in protection against metabolic dysregulation: Insights gained from model systems, especially the developing nervous system of zebrafish embryos. Free Radic Biol Med. 176:80-91. doi: 10.1016/j.freeradbiomed.2021.09.016.

Heise J., Schmitz T., Bührer C., Endesfelder S.. (2023). Protective Effects of Early Caffeine Administration in Hyperoxia-Induced Neurotoxicity in the Juvenile Rat. Antioxidants (Basel). 12(2):295. doi: 10.3390/antiox12020295.

Heuberger E., Hongratanaworakit T., Böhm C., Weber R., Buchbauer G. (2001). Effects of chiral fragrances on human autonomic nervous system parameters and self-evaluation. Chem Senses. 26(3):281-92. doi: 10.1093/chemse/26.3.281.

Iannuzzi C., Liccardo M., Sirangelo I. (2023). Overview of the Role of Vanillin in Neurodegenerative Diseases and Neuropathophysiological Conditions. Int J Mol Sci. 24(3):1817. doi: 10.3390/ijms24031817.

James, J.E. (2026). Maternal Caffeine Consumption and Pregnancy Outcomes. In: Bukiya, A.N., Ali, D.W. (eds) Molecular Mechanisms and Lifelong Consequences of Prenatal Exposure to Psychoactive Substances. Advances in Experimental Medicine and Biology, vol 1500. Springer, Cham. doi: 10.1007/978-3-032-12741-9_10.

JECFA (Joint FAO/WHO Expert Committee on Food Additives). (2005). Evaluation of certain food additives and contaminants: sixty-third report of the Joint FAO/WHO Expert Committee on Food Additives. Rome, Italy. WHO technical report series 928.

Jin Y., Wu H., Cohen E.M., Wei J., Jin H., Prentice H., et al. (2007). Genistein and daidzein induce neurotoxicity at high concentrations in primary rat neuronal cultures. J Biomed Sci. 14(2):275-84. doi: 10.1007/s11373-006-9142-2.

Kodama R., Okubo A., Araki E., Noda K., Ide H., Ikeda T. (1977a). Studies on d-limonene as a gallstone solubilizer. VII. Effects on development of mouse fetuses and offsprings (Jpn.). Oyo Yakuri. 13:863–873.

Kodama R., Okubo A., Sato K., Araki E., Noda K., Ide H. et al. (1977b). Studies on d-limonene as a gallstone solubilizer. IX. Effects on development of rabbit fetuses and offsprings (Jpn.). Oyo Yakuri. 13:885–898.

Kühne B.A., Puig T., Ruiz-Martínez S., Crous-Masó J., Planas M., Feliu L., et al. (2019). Comparison of migration disturbance potency of epigallocatechin gallate (EGCG) synthetic analogs and EGCG PEGylated PLGA nanoparticles in rat neurospheres. Food Chem Toxicol. 123:195-204. doi: 10.1016/j.fct.2018.10.055.

Kühne B.A., Teixidó E., Ettcheto M., Puig T., Planas M., Feliu L., et al. (2022). Application of the adverse outcome pathway to identify molecular changes in prenatal brain programming induced by IUGR: Discoveries after EGCG exposure. Food Chem Toxicol. 170:113506. doi: 10.1016/j.fct.2022.113506.

Patel R.P., Barnes S. (2010). Isoflavones and PPAR Signaling: A Critical Target in Cardiovascular, Metastatic, and Metabolic Disease. PPAR Res. 2010:153252. doi: 10.1155/2010/153252.

Peter B., Farkas E., Forgacs E., Saftics A., Kovacs B., Kurunczi S., et al. (2017). Green tea polyphenol tailors cell adhesivity of RGD displaying surfaces: multicomponent models monitored optically. Sci Rep. 7:42220. doi: 10.1038/srep42220.

Porciúncula L.O., Sallaberry C., Mioranzza S., Botton P.H., Rosemberg D.B. (2013). The Janus face of caffeine. Neurochem Int. 63(6):594-609. doi: 10.1016/j.neuint.2013.09.009.

Ravichandran C., Badgujar P.C., Gundev P., Upadhyay A. (2018). Review of toxicological assessment of d-limonene, a food and cosmetics additive. Food Chem Toxicol. 120:668-680. doi: 10.1016/j.fct.2018.07.052.

Salucci S., Ambrogini P., Lattanzi D., Betti M., Gobbi P., Galati C., et al. (2014). Maternal dietary loads of alpha-tocopherol increase synapse density and glial synaptic coverage in the hippocampus of adult offspring. Eur J Histochem. 58(2):2355. doi: 10.4081/ejh.2014.2355.

Santana A.B., Spelta L.E.W., Martinez-Sobalvarro J.V., Garcia R.C.T., Dos Reis T.M., Torres L.H. (2024). Prenatal caffeine consumption and neurobehavioral disorders - A systematic review. Reprod Toxicol. 125:108563. doi: 10.1016/j.reprotox.2024.108563.

Seyoum Tola F. (2024). The concept of folic acid supplementation and its role in prevention of neural tube defect among pregnant women: PRISMA. Medicine (Baltimore). 103(19):e38154. doi: 10.1097/MD.0000000000038154.

Shirpoor A., Salami S., Khadem-Ansari M.H., Minassian S., Yegiazarian M. (2009). Protective effect of vitamin E against ethanol-induced hyperhomocysteinemia, DNA damage, and atrophy in the developing male rat brain. Alcohol Clin Exp Res. 33(7):1181-6. doi: 10.1111/j.1530-0277.2009.00941.x.

Sownndhararajan K., Cho H., Yu B., Kim S. (2015). Effect of olfactory stimulation of isomeric aroma compounds, (+)-limonene and terpinolene on human electroencephalographic activity. Eur. J. Integr. Med. 7(6):561-566. doi: 10.1015/j.eujim.2015.08.006

Suzuki Y., Isemura M. (2001). Inhibitory effect of epigallocatechin gallate on adhesion of murine melanoma cells to laminin. Cancer Lett. 173(1):15-20. doi: 10.1016/s0304-3835(01)00685-1.

Tsuji M., Fujisaki Y., Okubo A., Arikawa Y., Noda K., Ide H., Ikeda T. (1975). Studies on *d*-limonene as a gallstone solubilizer. V. Effects on development of rat fetuses and offspring (Jpn.). Oyo Yakuri. 10:179–186.

Vijayalaxmi. (1980). Genetic effects of turmeric and curcumin in mice and rats. Mutat Res. 79(2):125-32. doi: 10.1016/0165-1218(80)90080-4.

Xu X., Zhang Z., Lin Y., Xie H. (2024). Risk of Excess Maternal Folic Acid Supplementation in Offspring. Nutrients. 16(5):755. doi: 10.3390/nu16050755.

Yang J., Jiang L.N., Yuan Z.L., Zheng Y.F., Wang L., Ji M., et al. (2008). Impacts of passive smoking on learning and memory ability of mouse offsprings and intervention by antioxidants. Biomed Environ Sci. 21(2):144-9. doi: 10.1016/S0895-3988(08)60020-3.

Yuede C.M., Olney J.W., Creeley C.E. (2013). Developmental neurotoxicity of alcohol and anesthetic drugs is augmented by co-exposure to caffeine. Brain Sci. 3(3):1128-52. doi: 10.3390/brainsci3031128.

Yun J. (2014). Limonene inhibits methamphetamine-induced locomotor activity via regulation of 5-HT neuronal function and dopamine release. Phytomedicine. 21(6):883-7. doi: 10.1016/j.phymed.2013.12.004.
