# Supplementary material for: Leveraging the developmental neurotoxicity in vitro battery: lessons learned interpreting assay outputs and integrating human exposure
Source: Front Toxicol. 2026 Sep 24;8:1919390. doi: 10.3389/ftox.2026.1919390 (PMC13645332; doi:10.3389/ftox.2026.1919390)
Supplement: Supplementary file 2 [file Supplementaryfile2.docx]

**Supplementary File 2: Solubility Limitations and Cytotoxicity in Range Finding Experiments**

This supplementary file provides detailed information on the solubility limitations and cytotoxicity observations encountered during the range finding and preparation of compounds for the DNT In Vitro Battery (DNT-IVB) assays.

## 1. Solubility Limitations

During preparation of stock solutions for the eight tested compounds (vitamin E/DL-α-tocopherol acetate, folic acid, genistein, caffeine, vanillin, epigallocatechin gallate [EGCG], curcumin, and (R)-(+)-limonene), solubility in the chosen solvent (dimethyl sulfoxide, DMSO) was assessed. Most compounds were initially targeted for stock solutions at 100 mM concentration. However, the following solubility limitations were identified:

- Folic Acid: Insufficient solubility at 100 mM; stock solution prepared at 20 mM.
- Curcumin: Insufficient solubility at 100 mM; stock solution prepared at 20 mM.
- Vitamin E (DL-α-tocopherol acetate): Insufficient solubility at 100 mM; stock solution prepared at 10 mM.

All other compounds were prepared at the intended 100 mM stock concentration, unless otherwise affected by cytotoxicity in subsequent range finding experiments. DMSO at a final concentration of 0.1% was used as the solvent control, as previous studies confirmed it does not affect cell viability or function in the Neurosphere or hNNF assays.

These solubility constraints directly influenced the highest testable concentrations in the assay dilution series and should be considered when interpreting negative results or lack of observable bioactivity at higher concentrations.

## 2. Cytotoxicity Observed in Range Finding Experiments

Preliminary range finding experiments were performed to establish non-cytotoxic concentration ranges for each compound prior to full-scale testing in the Neurosphere and hNNF assays. The following notable cytotoxicity observations restricted the upper concentration limits for certain compounds:

- Epigallocatechin gallate (EGCG): Exposure to 100 µM resulted in cytotoxicity and strongly reduced cell viability. The highest concentration tested in subsequent experiments was limited to 20 µM.
- Genistein: Exposure to 100 µM resulted in cytotoxicity and strongly reduced cell viability. The highest concentration tested in subsequent experiments was limited to 50 µM.

For all other compounds, range finding did not indicate cytotoxicity up to the solubility-limited or intended top concentrations.

## 3. Overview Table: Compound Preparation Constraints

| Compound | Stock Concentration Prepared | Highest Tested Concentration | Limiting Factor |
| --- | --- | --- | --- |
| Vitamin E (DL-α-tocopherol acetate) | 10 mM | 10 µM | Solubility |
| Folic Acid | 20 mM | 20 µM | Solubility |
| Curcumin | 20 mM | 20 µM | Solubility |
| Epigallocatechin gallate (EGCG) | 100 mM | 20 µM | Cytotoxicity |
| Genistein | 100 mM | 50 µM | Cytotoxicity |
| Caffeine | 100 mM | 100 µM | None |
| Vanillin | 100 mM | 100 µM | None |
| (R)-(+)-Limonene | 100 mM | 100 µM | None |

## 4. Implications for Data Interpretation

The above solubility and cytotoxicity limitations define the parameter space within which developmental neurotoxicity (DNT) effects could be reliably assessed for each compound. Where solubility or cytotoxicity precluded testing at higher concentrations, negative findings should be interpreted within the context of these technical limitations.
